# Supplementary material for: RiboGraph: an interactive visualization system for ribosome profiling data at read length resolution
Source: Bioinformatics. 2024 Jun 19;40(6):btae369. doi: 10.1093/bioinformatics/btae369 (PMC11197854; doi:10.1093/bioinformatics/btae369)
Supplement: btae369_Supplementary_Data [file btae369_supplementary_data.docx]

Supplemental Table 1.

| ***Software*** | ***Input File Format*** | ***RPF Analysis at Nucleotide Resolution*** | ***Availability of Source Code and Installation Instructions*** | ***Interactive*** |
| --- | --- | --- | --- | --- |
| *RiboGraph* | *.ribo* | *Yes* | *Yes* | *Yes* |
| *Shoelaces* | *.bam* | *No* | *Yes* | *Limited/No* |
| *RiboStreamR* | *.bam* | *Partial/Yes* | *Yes* | *Yes* |
| *riboviz2* | *.fastq* | *Yes* | *Yes* | *Yes* |
| *RiboToolkit* | *.fastq* | *Yes* | *No* | *Limited* |
| *Orfik* | *.fastq or .bam* | *No* | *Yes* | *No* |
| *riboFootPrinter* | *.sam* | *Yes* | *Yes* | *No* |
